# Supplementary material for: HIV-1 Vpr drives epigenetic remodeling to enhance virus transcription and latency reactivation
Source: bioRxiv. 2025 Jan 31:2025.01.31.635859. Preprint. [Version 1] doi: 10.1101/2025.01.31.635859 (PMC11838372; doi:10.1101/2025.01.31.635859)
Supplement: 1 [file NIHPP2025.01.31.635859v1-supplement-1.pdf]

bioRxiv preprint doi: https://doi.org/10.1101/2025.01.31.635859; this version posted January 31, 2025. The copyright holder for this preprint (which was not certified by peer review) is the author/funder, who has granted bioRxiv a license to display the preprint in perpetuity. It is made available under aCC-BY 4.0 International license.

Figure S1. Vpr-induced global epigenetic remodeling in immortalized and primary MDMs.

(**A**) Representative fluorescence microscopy images of histone marks in THP1 cells infected with indicated viruses.

(**B**) Representative fluorescence microscopy images of histone marks in primary MDM cells infected with indicated viruses.

Figure S2. Vpr-induced global epigenetic remodeling in HeLa cells.

(**A**) Representative fluorescence microscopy images and quantification of histone marks in HeLa cells infected with indicated viruses (n = 50).

(**B**) Flow cytometric analysis of DDR activation and histone marks in HeLa cells infected with indicated viruses.

(**C**) Immunoblot analysis of histone marks in HeLa cells infected with indicated viruses.

(**D** and **E**) Immunoblot analysis (D) and representative fluorescence microscopy images and quantification (E) of DDR activation in HeLa cells infected with indicated viruses.

Figure S3. Vpr mutant-induced DDR and histone modifications

(**A**) Quantification of DDR activation following infection of THP1 cells with Vpr_{WT} or mutant viruses (n = 50).

(**B**) Quantification of histone marks in HeLa cells infected with indicated viruses (n = 50).

(**C**) Representative fluorescence microscopy images and RT-PCR analysis of DCAF1 expression in HeLa cells transfected with DCAF1 shRNAs.

bioRxiv preprint doi: https://doi.org/10.1101/2025.01.31.635859; this version posted January 31, 2025. The copyright holder for this preprint (which was not certified by peer review) is the author/funder, who has granted bioRxiv a license to display the preprint in perpetuity. It is made available under aCC-BY 4.0 International license.

Figure S4. Vpr modulation of HIV promoter activity

(**A**) Flow cytometric analysis of HIV LTR activity in HeLa (top) and THP1 (bottom) cells infected with indicated viruses in the presence or absence of DDR inhibitors.

(**B**) Quantification of HIV LTR activity in primary MDMs infected with Vpr_{WT} virus in the presence or absence of DDR inhibitors.

(**C**) Quantification of histone marks in uninfected HeLa cells in the presence or absence of HDAC inhibitors (n = 50).

(**D**) Diagram displaying the establishment of the HeLa latency model H-Lats (left) and representative live cell fluorescence microscopy images (right) of H-Lat GFP expression when infected with indicated viruses.

(**E**) Flow cytometric analysis of H-Lat GFP expression when infected with indicated viruses.
